# Imaging the Three-Dimensional Ionospheric Structure with a Blob Basis Functional Ionospheric Tomography Model

**DOI:** 10.3390/s20082182

**Published:** 2020-04-12

**Authors:** Debao Wen, Dengkui Mei, Yanan Du

**Affiliations:** 1 School of Geographical Science, Guangzhou University, Guangzhou 510006, China; yndu@gzhu.edu.cn; 2 School of Traffic and Transportation Engineering, Changsha University of Science & Technology, Changsha 410004, China; mei_dk@163.com

**Keywords:** ionospheric tomography, total electron content, model, ionospheric electron density

## Abstract

A new ionospheric tomography model is presented in this work. In the new model, the traditional voxel basis function is replaced by the blob basis function. Due to the overlapping nature of their rotational symmetric basis functions, the new model introduces certain weighting from nearby tomographic spherical blobs. To confirm the feasibility of the new tomography model, a numerical simulation scheme is devised, and the simulation demonstrates that the reconstructed quality of the blob basis tomographic model is higher than that of the voxel basis tomographic model. Meanwhile, the variable blob radius is adopted in order to improve the efficiency of the new model. Finally, the new ionospheric tomography model is applied to reconstruct the temporal-spatial distribution of ionospheric electron density using actual global navigation satellite system observations. The comparisons between the tomographic profiles and those obtained from ionosonde data further demonstrate the reliability and the superiority of the new ionospheric tomography model.

## 1. Introduction

The ionosphere is the upper atmosphere of the earth which is ionized by the radiation of the sun’s energetic particles. Vertical total electron content (VTEC) and electron density are the two important physical parameters to study the temporal-spatial structure of the ionosphere. Ionospheric electron density (IED) distributions typically vary with seasons, solar activity, and geographic location [1]. In general, VTEC values are obtained from the single-layer ionospheric model in which all electrons are assumed to be concentrated on a thin layer with a fixed altitude. Therefore, VTEC can be used to image two-dimensional structure of the ionosphere in the latitude and longitude cross section, but it ignores the vertical variation of the ionospheric structure [2,3]. To grasp the ionospheric vertical structure, Austen et al. [4] introduced computerized tomography technique to reconstruct two dimensional ionospheric images in the latitude and height cross section. However, it cannot reflect the longitudinal variations of the ionosphere because the ground receiver is arranged along a fixed longitudinal chain. It is necessary to know and map the temporal-spatial variations of three-dimensional ionosphere structure for high-accuracy ionospheric delay correction. Kunitsyn et al. [5] confirmed that it was possible to reconstruct three-dimensional IED distributions using the simulated high orbit satellite data. From then on, three-dimensional computerized ionospheric tomography has become a popular reconstruction technique of IED distributions. Theoretical and experimental research has been thoroughly carried out in order to reconstruct IED images by using global navigation satellite System (GNSS) data. Mitchell and Spencer [6] studied a three-dimensional time dependent algorithm to image the ionosphere based on global positioning system (GPS) data. Ma et al. [7] introduced neural network to map the three-dimensional ionospheric structure by assimilate the GPS data and the ionosonde data; Lee et al. [8] reconstructed the three-dimensional images of ionospheric variability using a dense GPS receiver array; Xiao et al. [9] investigated the abnormal distribution of the IED during November 2004 super-storm by 3D tomographic reconstruction from international GNSS service, low earth orbit and GPS observations. Yao et al. [10] proposed a hybrid regularization method to resolve the ill-posed problem of GPS ionospheric tomography. Zheng et al. [11] investigated ionospheric tomography technique based on variable pixel height.

In ionospheric tomography studies, most scholars usually used the voxel basis tomographic model to reconstruct the IED distributions [12,13,14,15,16,17,18]. In that model, the selected function is the voxel basis function and the image region is divided into non-overlapping cubic voxels. It results in that the model cannot effectively reconstruct the continuity of IED distributions. The imaging quality of IED distributions is relatively poor. For image reconstruction, Matej and Lweitt [19] validate the blob basis function is superior to the voxel basis function by using simulation experiment, and high-resolution reconstructed images are obtained. Considering the advantages of the blob basis function, we attempt to use the blob basis function to reconstruct ionospheric electron density distribution. The ionospheric space is divided into overlapping spherical blobs in order to resolve the above problem, and blob basis function is innovatively introduced to replace traditional voxel basis function in this work. We can efficiently reconstruct the higher-quality IED images due to the overlapping nature of its rotational symmetric basis function by using the blob basis ionospheric tomographic model. To validate the feasibility and the superiority of the blob basis ionospheric tomographic model, numerical testing and actual experiments are carried out. The experimental results show that the reconstructed IED images of the blob basis model are usually better than those reconstructed from the traditional voxel basis model, and the efficiency of the new tomographic model is higher than the voxel basis model.

## 2. Methodology

In general, ionospheric slant total electron content (STEC) is the linear integration of IED along a ray propagation path. It is written as

(1) $$STEC_{i}=\int_{S_{i}}^{}N_{e}\left(\vec{p}\right)ds\;\left(i=1,2,\cdots ,m\right),$$

where *s_i_* is the *i*th ray propagation path. $N_{e}\left(\vec{p}\right)$ is the IED distribution along the ray paths. $\vec{p}$ is the position vector. *m* represents the number of STEC measurements.

To simplify the inversion of the IED, the reconstructed ionospheric space is usually discretized into some small voxels, and then Equation (1) can be represented as

(2) $$STEC_{i}=\sum_{j=1}^{n}Ne_{j}\int_{S_{i}}^{}b_{j}\left(\vec{p}\right)ds=\sum_{j=1}^{n}A_{ij}Ne_{j},$$

where *A_ij_* is the distance of the ith ray traversing jth volume element. *b_j_* is the selected basis function. *Ne_j_* is the electron density in *j*th volume element. *n* is the number of the volume elements. Taking the discretization error and observation error into account, Equation (2) can be represented as

(3) $$STEC_{i}=\sum_{j=1}^{n}A_{ij}Ne_{j}+E_{i}.$$

Equation (3) can be generally written in simple matrix notation as

(4) Y=AX+E.

*Y* is a column vector of m known *STEC* measurements. *A* is a coefficient matrix. *X* is the column vector consisting of the unknown IED. *E* is a vector associated with discretization errors and measurement noises.

**Figure 1:**
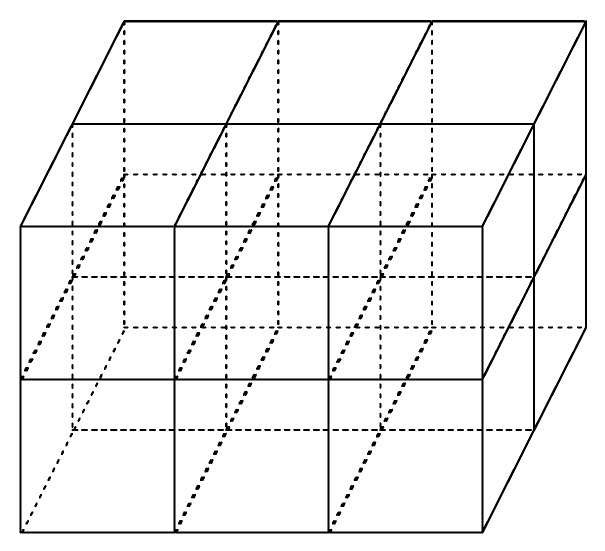
Sketch map of the discrete voxels partition.

From Equation (2), it can be seen that the selection of basis function is very important for the linearization. As shown in Figure 1, the imaging area is first divided into some non-overlapping discrete voxels when the voxel basis tomographic model is used. The chosen basis function is the voxel index function, which is a piecewise function.

(5) $$b_{j}\left(\vec{r}\right)=\{\begin{array}{ll} 1 & \left(\vec{r}\in voxel\right)\\ 0 & \left(otherwise\right) \end{array}.$$

Using the voxel index function, the *A_ij_* in Equation (2) is assumed to be the crossing distance of the ith straight ray path traversing jth the voxel in order to simplify the computation. This is inconsistent with the real situation of ray propagation. However, the accuracy of the crossing distance is very important to obtain high-accuracy IED distributions.

**Figure 2:**
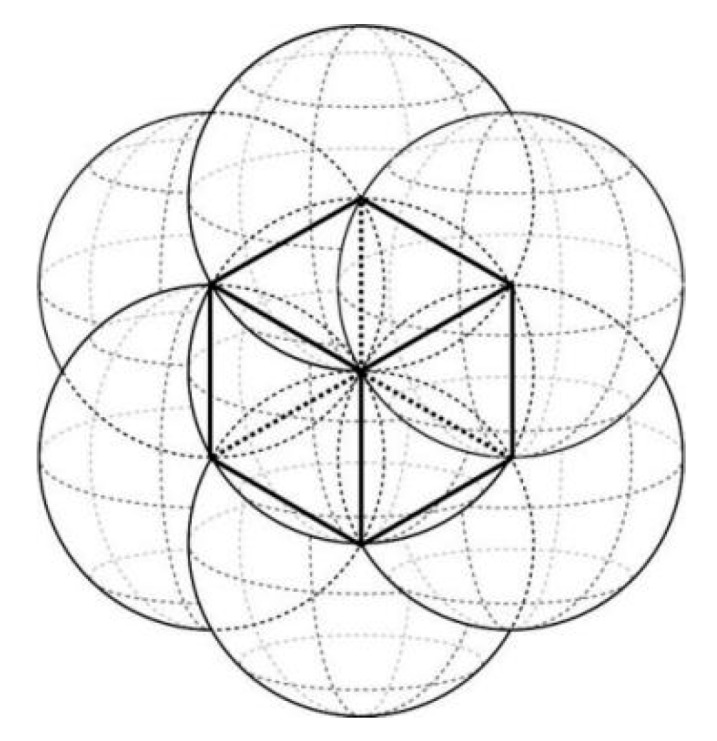
Diagram of the spherical blobs division.

To overcome the disadvantages of voxel representation, the traditional voxel basis ionospheric tomography model is replaced by the blob basis ionospheric tomography model. Different from the method of traditional voxel partition, the ionospheric space is discretized into some overlapping spherical blobs when the blob basis reconstruction model is used to reconstruct IED distributions. Figure 2 illustrates the sketch map of spherical blob division.

In the new model, the basis function is constructed using the generalized Kaiser-Bessel (KB) window functions

(6) $$b\left(r\right)=\{\begin{array}{ll} \frac{{\left(\sqrt{1-{\left(r/a\right)}^{2}}\right)}^{h}I_{h}\left(\alpha \sqrt{1-{\left(r/a\right)}^{2}}\right)}{I_{h}\left(\alpha \right)} & \left(0\leq r\leq a\right)\\ 0 & \left(otherwise\right) \end{array},$$

where r is the radial distance from the blob center, which is associated with the location of the cross point of the ray traversing the spherical blob. *I_h_* denotes the modified Bessel function of h orders [20], which controls the continuity condition of the blob boundary. *a* is the radius of the spherical blob, and α is the non-negative real number controlling the shape of the blob.

According to the above selections of basis function and the spherical blob, the series crossing point can be found when the ray traversing the spherical blob, and then the real crossing distance in Equation (2) can be obtained. This means that the integration path is closer to the real state of ray propagation.

## 3. Results and Discussions

### 3.1. Numerical Simulation Experiment

The calculation of high accuracy *STEC* is very important to ensure the reconstructed accuracy of the IED. Although the absolute *STEC* can be obtained by using the differential pseudoranges, its accuracy is usually low. In general, the high accuracy differential phases can be applied to derive the *STEC*. However, the *STEC* represents the relative changes of ionospheric *TEC* due to the existence of the ambiguity in the phase measurements. To obtain the high absolute *STEC*, an extra term B_L_ is introduced in this work. It can be formulated as follows [7,21,22]

(7) $$STEC=STEC_{\Phi }+B_{L}.$$

If N measurements are obtained during a satellite pass, the extra term *B_L_* can be written as

(8) $$B_{L}=\sqrt{\frac{\sum_{i=1}^{N}{\left(STEC_{P_{i}}-STEC_{\phi_{i}}\right)}^{2}}{N}}.$$

The method described by Blewitt [23] is used to preprocess the selected GNSS observations. The L4 combination is formed to remove the effects of geometry, clock bias, and tropospheric delay error. In general, the instrumental bias of satellite and receiver is usually stable in one day. In this work, the instrumental bias is fitted using the least square technique.

**Figure 3:**
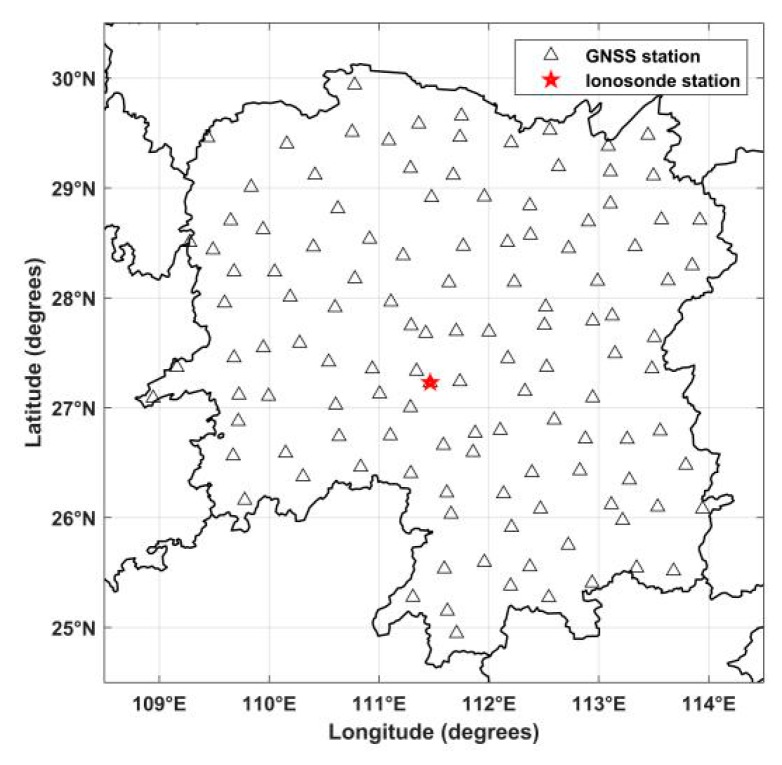
Distribution of continuously operating reference system GNSS stations and ionosonde station in Hunan province.

To validate the feasibility and the superiority of the new tomographic model, a numerical simulation experiment is carried out in this work. For this purpose, the projection matrix *A* in Equation (4) can be created using the coordinates of the observed GNSS satellites and 124 ground stations from the continuously operating reference system (CORS) of Hunan province in China. The distribution of CORS GNSS stations and the ionosonde station are shown in Figure 3.

Using the internation reference ionospheric (IRI) model, such as IRI 2016 model, the IED true value *X_sim_* can be obtained. The simulated *STEC* without noise can be computed using the following equation.

(9) $$Y_{sim}=AX_{sim}.$$

To make the simulated *STEC* closer to its actual situation, the simulated *STEC* vector *Y_sim_* is added a small amount of random white noise *E_sim_*. The added amount equals the 5% of the simulated *STEC* data in this work. The *STEC* vector with random white noise can be then represented as

(10) $$Y_{noise}=Y_{sim}+E_{sim}.$$

To ascertain the parameters of blob basis function and compare characteristics of the two tomographic models, the hybrid reconstruction algorithm [24], which combines the truncated singular value decomposition (TSVD) with algebraic reconstruction technique (ART), is introduced to perform the two tomographic models, and the true IED value is simulated by using the IRI 2016 model. Since the geomagnetic activity is quiet, the selected data is the GNSS observations on June 15, 2015. The simulated time period is 00:00 UT–00:30 UT. The latitude ranges from 24° N to 30° N. The longitude varies from 109° E to 114° E, and the height ranges from 100 km to 1000 km.

For the simulation experiment, the key problem is to determine the parameters *h*, *α*, and *a* that affect the quality and efficiency of the imaging reconstruction. Whether the observation data is sufficient or not, Matej and Lweitt [19] have verified that the blob basis function and the first derivative are continuous when *h* equals 2. For the blob basis tomographic model, the selection of the parameters of α and *a* is different from the imaging reconstruction of all view angle. Considering the computational efficiency and the electron mainly concentrating on the height range from 200 km to 500 km, the variable parameter *a* is variable in different height ranges. To define the two important parameters, some schemes are devised, and the error statistic results of the different schemes are given in Table 1.

From Table 1, it can be seen that the reconstructed errors of scheme 2 is minor compared to other schemes in a different height range. In the following simulation experiments, the parameters *α* and *a* of scheme 2 are adopted, and the unit of *a* is kilometer. Therefore, the ionospheric space is discretized using scheme 2.

**Figure 4:**
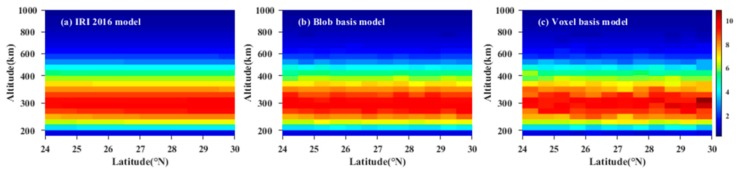
Comparison between the reconstructed IED distributions of the two models and those obtained from IRI 2016 model. The unit of IED is 10^11^ el/m^3^.

Figure 4 compares the reconstructed results of two models with the true value. From Figure 4, it can be seen that the reconstructed image of the blob basis tomographic model coincides with that obtained from IRI 2016 model. It demonstrates that the blob basis functional model is feasible to reconstruct the IED distributions. Meanwhile, Figure 4 also shows that the reconstructed IED distributions of the blob basis tomographic model have better agreement with the simulated true values of IED obtained from IRI 2016 model.

**Figure 5:**
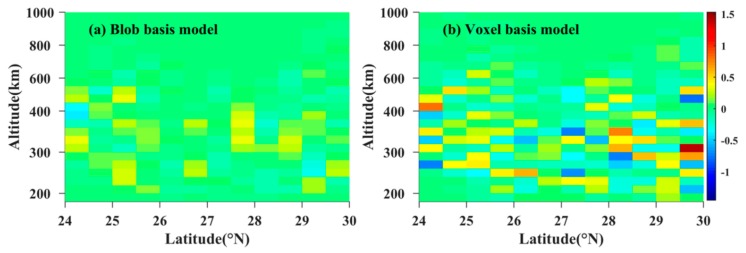
Comparison of the IED reconstructed errors between blob basis model and voxel basis model. The unit is 10^11^ el/m^3^.

Figure 5 illustrates the contour maps of the reconstructed errors of the two tomographic models. In Figure 5a, the maximum absolute error of IED reconstruction is 3.3 × 10^10^ el/m^3^, and the root mean square error (RMSE) is 1.05 × 10^10^ el/m^3^. However, for the voxel basis tomographic model, the maximum absolute error of IED reconstruction is 1.41 × 10^11^ el/m^3^, and the RMSE is 4.36 × 10^10^ el/m^3^. The comparison of the reconstructed errors validates that the accuracy of the blob basis model is higher than that of the voxel basis model. These facts confirm that the blob basis tomographic model is superior to the voxel basis tomographic model.

To test the convergence speed of the blob basis tomographic model, different blob divisions scheme is devised. One is the discretized space adopted in the simulation experiment, and the other scheme is the fixed radius of the blob, which is 15 km. In this work, the iteration convergence condition is defined as follows

(11) $$S=\frac{{∥x_{0}-x_{1}∥}_{2}}{{∥x_{1}∥}_{2}},$$

where *x*_0_ is the initial IED value of each spherical blob, which is roughly given by Truncated singular value. *x*_1_ represent the final IED solution vector of each spherical blob.

**Figure 6:**
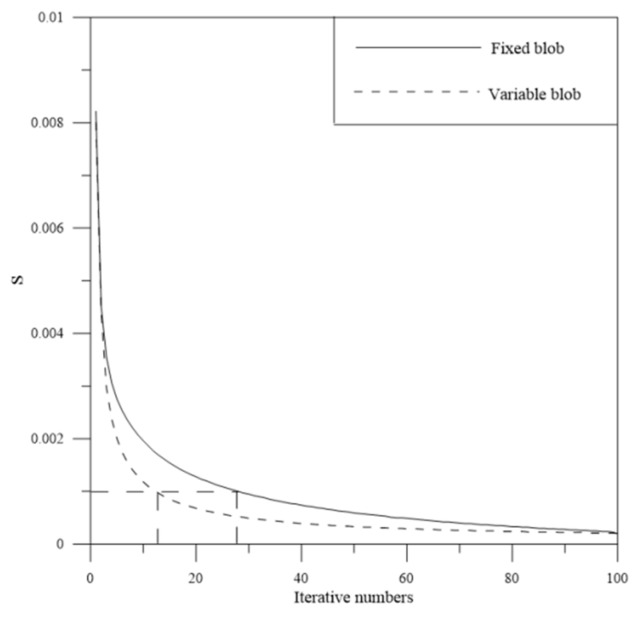
Convergence comparison of the blob basis model based on two different discretized scheme.

In the simulation, the convergence of the iteration is terminated when *S* < 10^−3^. Figure 6 shows the variation of the convergence speed. From Figure 6, we can see that the convergence is terminated when the iterative numbers is 12, and the convergence speed of the variable blob basis model is faster than that of the fixed blob basis model. It validates that the variable blob basis model is an efficient tomographic model.

### 3.2. Tomography Reconstruction of IED Using Actual GNSS Data

To further validate the reliability of the new tomographic model, it is necessary to reconstruct the IED distributions by combining the new model with real GNSS observations. In this work, the reconstructed geographic region and the discrete intervals are the same as those of the simulated experiment. The GNSS data of 124 ground stations are obtained from the CORS network in Hunan province. The sample interval of the GNSS data is 30 seconds and the elevation mask angle is 15°. The method proposed by Blewitt [22] is used to preprocess the selected GNSS data. Using the new model and the preprocessed GNSS data, the time-series variations of the three-dimensional IED distributions are effectively reconstructed and analyzed on 15 June 2015.

**Figure 7:**
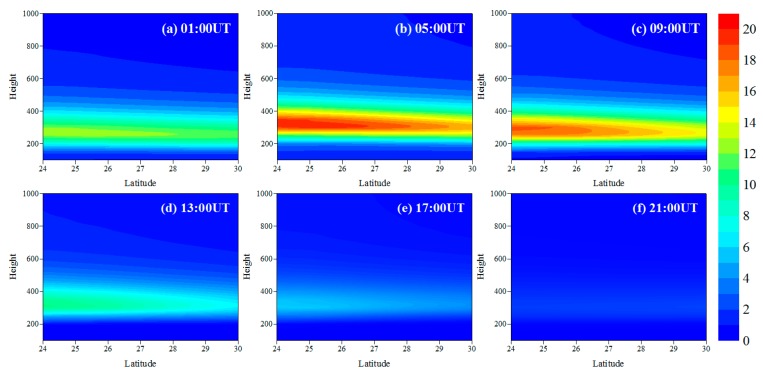
Time-series images of the reconstructed IED distributions on June 15, 2015, in the cross section of latitude and height at 111.5° E. The recording time for each panel is given at the top right corner of each panel. The unit of IED and height is 10^11^ el/m^3^ and km, respectively.

Figure 7 shows the diurnal variations of IED distributions in the cross section of latitude and height. It can be seen that the IED varies from small to large between 01 UT and 05 UT, and then the IED varies from large to small as the time evolves, which is consistent with the normal change laws in daytime and nighttime over Hunan province. At 05 UT, the IED reaches the maximum that is 1.95 × 10^12^ el/m^3^. However, the minimum of IED occurs at 21 UT, the value 2.82 × 10^11^ el/m^3^. Comparing all panels in Figure 7, it can be seen that the peak height of the IED gradually rises from 260 km to 350 km between 01 UT and 05 UT, and then it falls to 290 km between 09 UT and 21 UT. It roughly reflects the vertical variation characteristics of the ionosphere in the reconstructed geographical region.

**Figure 8:**
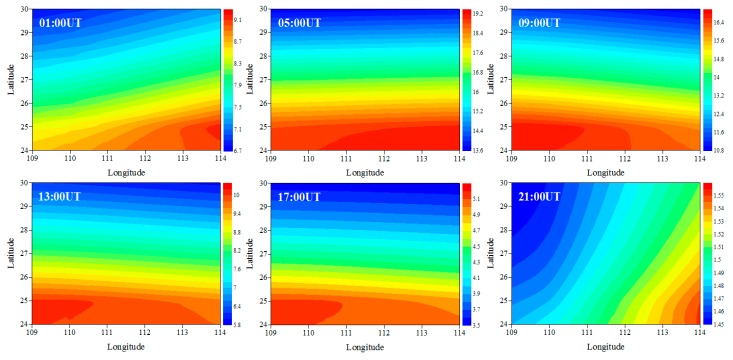
Tomographic images of IED on June 15, 2015, in the longitude and latitude plane at 350 km. The recording heights for each panel are given at the top left corner of each panel. The unit of the IED is 10^11^ el/m^3^.

Figure 8 illustrates the time-series variations of IED with longitude and latitude at 350 km on June 15, 2015. It can be seen that the IED in eastern Hunan is higher than that in western Hunan from 01 UT to 05 UT. With the time elapses, the IED in western Hunan gradually increases. The IED in western Hunan is higher than that in the eastern Hunan from 09 UT to 17 UT. At 21 UT, the IED in the eastern Hunan is higher than that in western Hunan. From Figure 7 and Figure 8, we can also see that there are larger differences between the characteristics of the ionosphere in mid-latitude and low-latitude regions, and the IED values over northern Hunan are smaller than those over southern Hunan as a whole. This indicates a strong correlation between the variation of IED and latitude.

**Figure 9:**
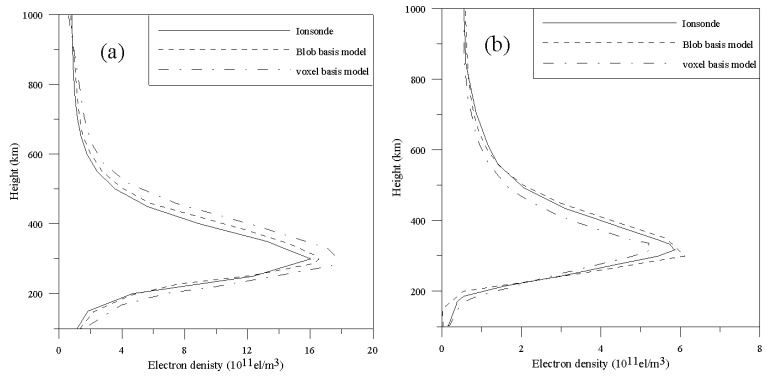
Comparison of the reconstructed IED profiles of the two models with the corresponding density profiles measured by ionosonde station at Shaoyang. (**a**) 05 UT; (**b**) 13 UT.

Figure 9 illustrates the comparison of the reconstructed IED profiles using the two models with that obtained from ionosonde data recorded by Shaoyang Station at 09 UT and 17 UT. From Figure 9, it can be seen that the reconstructed IED profiles of the blob basis tomographic model have better agreement with that obtained from ionosonde data than those of the voxel basis tomographic model as a whole. The same conclusion can also be draw from other reconstructed IED vertical profiles. The bottom IED profile shows that the peak height of the reconstructed profiles of the blob-based model is lower than that of ionosonde. The reconstructed results further validate that the blob basis tomographic model is superior to the voxel basis tomographic model. It can reconstruct the IED distributions using higher accuracy.

The Kp index of geomagnetism is more than 5 betwee 15 UT on 22 June 2015, and 12 UT on 23 June 2015. In this time period, the Kp indexes range from 5 to 8. This indicates that a strong geomagnetic storm happened in this time period. Using the blob basis tomographic model, we calculate the differences between the storm-time IED and the reference values, which equal the 3-day IED average values before the storm occurrence.

**Figure 10:**
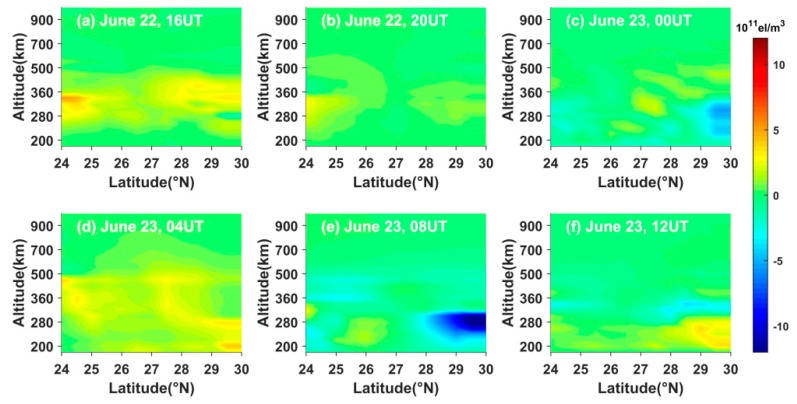
Time-series variation of two-dimensional differential IED along the longitude of 111° E on 22 and 23 June 2015.

Figure 10 shows the latitude-altitude variations of the differential IED along the longitude of 111° E on 22 and 23 June 2015. From Figure 10, it can be seen that the weak positive phase storm appeared between 280 km and 360 km at 16 UT on 22 June 2015, and then the intensity of the positive phase storm decreases with time. Then the IED values are almost unchanged at 20 UT on 22 June and 00 UT on 23 June, except for a small decrease ranging from 29° N to 30° N at the altitude of about 280 km. As the storm developed, the positive phase storm covering a wider height range appeared at 04 UT on 23 June. Afterwards, the strong negative phase storm occurred from 260 km to 300 km in the northern Hunan, where the IED values obviously decreased. Then the negative storm almost disappeared, and the IED values showed a slight increase from 200 km to 280 km in the northern Hunan province at 12 UT on 23 June.

## 4. Conclusions

A new ionospheric tomography model is presented. The new model uses the overlapping blobs to replace the voxels in the traditional voxel basis model. To validate the new tomographic model, two tests are carried out. The reliability of the new model is first confirmed by a simulated experiment in which the IED distributions are generated from the IRI 2016 model. The simulated test shows that the new model is superior to the voxel basis ionospheric tomography model. Finally, the new model is further introduced to reconstruct the three dimensional IED distributions in a geomagnetically quiet day. The temporal and spatial variation characteristics of the three dimensional ionospheric structure are carefully investigated. The profiles obtained from the new tomographic model have better agreement with ionosonde than those reconstructed from the voxel basis tomographic model. The fact shows that the reconstructed accuracy of blob basis tomographic model is higher than that of the voxel basis tomographic model. The test results show that the new model is effective for the tomographic reconstruction of IED.

Although the new model is successfully applied to reconstruct three-dimensional IED distributions in the geomagnetically quiet day, it can also be extended to investigate the large-scale ionospheric disturbance under the condition of a magnetic storm and pre-earthquake in the further work. Using the new model, we can know and map the details of ionospheric anomalies caused by these special phenomena. In addition, the blob basis tomography can be exploited in other fields, such as medical science and seismic science.

## Figures and Tables

**Table 1:** Statistics of the reconstructed IED error using different schemes of parameters selection.

Range of Height (km)	Scheme		Average IED Errors (10^11^ el/m^3^)	Root Mean Square Error (10^11^ el/m^3^)
500–1000	1	*a* = 50, *α* = 3.0	0.14	0.10
	2	*a* = 50, *α* = 3.5	0.08	0.07
	3	*a* = 50, *α* = 4.0	0.13	0.12
200–500	1	*a* = 15, *α* = 3.0	0.47	0.43
	2	*a* = 15, *α* = 3.5	−0.18	0.15
	3	*a* = 15, *α* = 4.0	0.38	0.40
100–200	1	*a* = 50, *α* = 3.0	0.26	0.23
	2	*a* = 50, *α* = 3.5	0.15	0.10
	3	*a* = 50, *α* = 4.0	−0.20	0.16
